# Very Low-Efficiency Droop in 293 nm AlGaN-Based Light-Emitting Diodes Featuring a Subtly Designed p-Type Layer

**DOI:** 10.3390/molecules27217596

**Published:** 2022-11-05

**Authors:** Mu-Jen Lai, Yi-Tsung Chang, Shu-Chang Wang, Shiang-Fu Huang, Rui-Sen Liu, Xiong Zhang, Lung-Chien Chen, Ray-Ming Lin

**Affiliations:** 1 Jiangxi Yuhongjin Material Technology Co., Ltd., Fuzhou 344100, China; 2 Department of Physics, School of Science, Jimei University, Xiamen 361021, China; 3 Changshu Institute of Technology, College of Electronics and Information Engineering, Changshu 215500, China; 4 Department of Otolaryngology-Head and Neck Surgery, Chang Gung Memorial Hospital, Taoyuan 33302, Taiwan; 5 Department of Public Health, Chang Gung University, Taoyuan 33302, Taiwan; 6 Guangdong Institute of Semiconductor Micro-Nano Manufacturing Technology, Foshan 528200, China; 7 Advanced Photonics Center, School of Electronic Science and Engineering, Southeast University, Nanjing 210096, China; 8 Department of Electro-Optical Engineering, National Taipei University of Technology, Taipei 10608, Taiwan; 9 Department of Electronic Engineering and Institute of Electronics Engineering, Chang Gung University, Taoyuan 33302, Taiwan

**Keywords:** AlGaN, MOCVD, light-emitting diode, efficiency droop, external quantum efficiency

## Abstract

This paper reports an AlGaN-based ultraviolet-B light-emitting diode (UVB-LED) with a peak wavelength at 293 nm that was almost free of efficiency droop in the temperature range from 298 to 358 K. Its maximum external quantum efficiencies (EQEs), which were measured at a current density of 88.6 A cm^–2^, when operated at 298, 318, and 338 K were 2.93, 2.84, and 2.76%, respectively; notably, however, the current droop (J-droop) in each of these cases was less than 1%. When the temperature was 358 K, the maximum EQE of 2.61% occurred at a current density of 63.3 A cm^–2^, and the J-droop was 1.52%. We believe that the main mechanism responsible for overcoming the J-droop was the uniform distribution of the concentrations of injected electrons and holes within the multiple quantum wells. Through the subtle design of the p-type AlGaN layer, with the optimization of the composition and doping level, the hole injection efficiency was enhanced, and the Auger recombination mechanism was inhibited in an experimental setting.

## 1. Introduction

Severe acute respiratory syndrome coronavirus 2 (SARS-CoV-2) and COVID-19 have been stressing global healthcare [1,2] and economic systems [3]. The germicidal effect exerted by AlGaN-based deep-ultraviolet (DUV) light-emitting diodes (LEDs) displaying emission wavelengths of less than 315 nm has attracted much attention due to it being able to successfully inactivate COVID-19 [4,5,6,7]. Since the implementation of the Minamata Convention on Mercury, DUV-LEDs in particular have shown great potential in medical and agricultural applications when emitting light at wavelengths in the range of 285–315 nm [8,9,10,11,12,13]. For example, DUV-LEDs are suitable for the production of vitamin D_3_. In human skin, vitamin D_3_ production depends on several factors, including the person’s age, the time of day, the latitude and altitude, the season, the area of exposure, and the degree of skin pigmentation, with ultraviolet-B (UVB) wavelengths between 290 and 300 nm being most efficient [7,14,15,16,17,18,19,20,21,22]. Kalajian et al. [14] found that light at a wavelength of 293 nm was 2.4 times more efficient at producing vitamin D_3_ in human skin than light from the sun was, as measured according to the exposure time. This radiation penetrates the skin and is absorbed by 7-dehydrocholesterol (7-DHC) to form *cis-cis* previtamin D_3_, which undergoes isomerization to produce more thermodynamically stable vitamin D_3_. Several reports have revealed that the hole injection efficiency can be improved in AlGaN-based DUV-LEDs through the functionalization of the p-AlGaN electron-blocking layer (EBL) [7,23,24,25,26,27,28]. AlGaN-based DUV-LEDs that operate nearly free of efficiency droop have also been prepared [23,24,25]. Usman et al. [23] found through numerical analysis that the efficiency droop at 500 A cm^–2^ decreased from 42 to 7% when using an optimized undoped-AlGaN final quantum barrier, a p-type multiple quantum barrier EBL, and an Al composition-graded p-AlGaN hole injection layer in their UVB-LED structure. Jia et al. [24] demonstrated, through simulation, that the efficiency droop at an injection current of 120 mA decreased remarkably, from 35 to 8.9%, when employing a band-engineered quantum barrier without a p-type EBL. Zhang et al. [25] reported a system in which the efficiency droop was approximately 4% at a current density of 110 A cm^–2^, despite numerical calculations suggesting that it would almost be free of efficiency droop. Moreover, current droop (J-droop) and temperature droop (T-droop) processes, which are common with visible-light LEDs, are also severe problems in AlGaN-based UVB-LEDs, even for emission wavelengths of less than 280 nm [29,30,31,32,33,34,35,36]. In general, direct transitions between the conduction and valence bands are the key to enhancing the internal quantum efficiencies of multiple quantum wells (MQWs) when developing LEDs. Nevertheless, the distributions of the concentrations of electrons and holes in MQWs and the values of *K* of the electrons and holes of transition pairs should be the same. The biggest challenge when fabricating AlGaN-based UVB LEDs is the low doping efficiency of the p-AlGaN layer at high Al contents. Thus, we have previously suggested that one of the main reasons for the J-droop in AlGaN-based UVB LEDs is the insufficient conductivity of the neutral region of the p-AlGaN layers, with the number of holes injected into the MQWs resulting in a mismatch of the quasi-charge-neutral conditions [37]. To overcome the efficiency droop at a higher current density (>100 A cm^–2^), it is necessary to a subtly design the p-layer to improve the hole injection efficiency and the uniformity of the hole concentration within the well region of the MQWs, thereby avoiding Auger recombination as a result of a mismatch in the hole and electron concentrations. Nevertheless, only a few recent reports have appeared that have simultaneously discussed the phenomena of T-droop, J-droop, and current-induced degradation in AlGaN-based 293 nm UVB-LEDs [38,39,40,41,42,43]. Thus, in this study, we fabricated an AlGaN-based 293 nm UVB-LED and examined its electroluminescence (EL) characteristics over the temperature range from 298 to 358 K and at forward current densities (*J*_f_) in the range of 12.7–113.9 A cm^–2^ to obtain insight into the behavior of the EL emission and the efficiency droop. Furthermore, from an evaluation of the aging lifetimes of the 293 nm UVB-LEDs, we estimated that their performance was close to the specifications for commercial solid-state lighting products.

## 2. Materials and Methods

**Figure 1:**
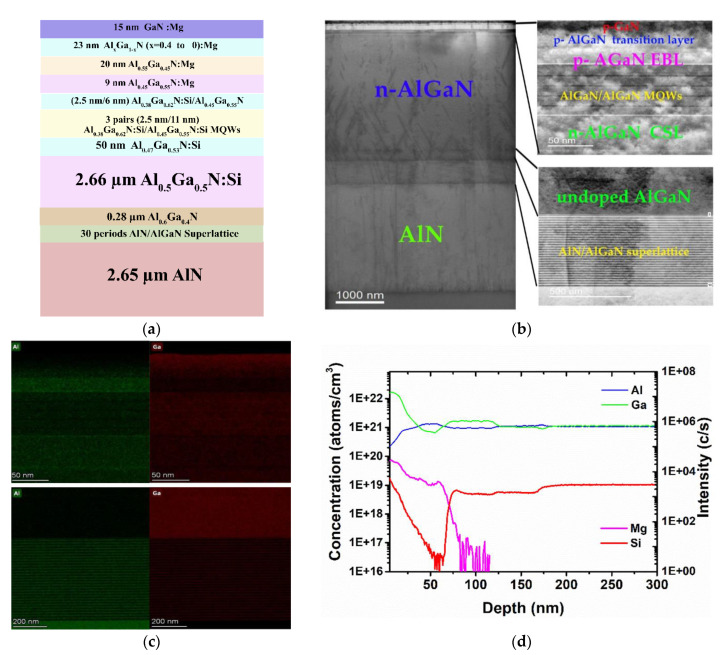
(**a**) Schematic representation of the heterostructure of the investigated 293 nm AlGaN-based UVB-LED. (**b**) TEM image of the 293 nm AlGaN-based UVB-LED structure. Insets: (top) Enlarged TEM image revealing the MQW and p-layer structures; (bottom) enlarged TEM image revealing the AlN layer and superlattice structures; (**c**) (top) EDX mapping image revealing the variable composition of Al and Ga in the MQW and p-layer structures; (bottom) EDX mapping image revealing the variable composition of Al and Ga in the undoped AlGaN and superlattice structures. (**d**) SIMS depth profiles of Mg, Si, Al, and Ga atoms in the 293 nm AlGaN-based UVB-LED.

Figure 1a displays a schematic representation of the 293 nm AlGaN-based UVB-LED, which was prepared using low-pressure metal–organic chemical vapor deposition (LP-MOCVD). Trimethylaluminum, trimethylgallium, silane, bis(cyclopentadienyl)magnesium, and ammonia were used as the Al, Ga, Si, Mg, and N sources, respectively. First, an AlN layer that had a thickness of 2.65 μm was deposited onto a 2-inch (0001)-oriented sapphire substrate through an AlN buffer layer that was 4.7 nm in thickness. Next, a strain-relieving interlayer that was 0.55 μm in thickness and consisting of 30 periods of an AlN/AlGaN superlattice with an equivalent Al composition of 0.72 was grown on the AlN layer. A layer of undoped Al_0.6_Ga_0.4_N that was 0.28 μm in thickness was then grown on the superlattice interlayer. Subsequently, a Si-doped n-Al_0.5_Ga_0.5_N layer that was 2.66 μm in thickness was grown as the n-contact layer. After that, a current-spreading layer (CSL) of Si-doped Al_0.47_Ga_0.53_N that was 50 nm in thickness was grown on the n-contact layer. The active region of the MQWs included three pairs of Si-doped Al_0.38_Ga_0.62_N quantum wells (QWs) that were 2.5 nm in thickness and Si-doped Al_0.45_Ga_0.55_N quantum barriers (QBs) that were 11 nm in thickness, followed by a Si-doped Al_0.38_Ga_0.62_N QW that was 2.5 nm in thickness and an undoped Al_0.45_Ga_0.55_N QB that was 6 nm in thickness. A structure consisting of Mg-doped Al_0.45_Ga_0.55_N that was 9 nm in thickness and Mg-doped p-Al_0.55_Ga_0.45_N that was 20 nm in thickness was grown as the EBL on the undoped Al_0.45_Ga_0.55_N QB. Subsequently, a Mg-doped p-AlGaN layer that was 23 nm in thickness with the Al content grading down from 40 to 0% was grown on the EBL as the transition layer. Finally, a Mg-doped p-GaN layer that was 15 nm in thickness was deposited, serving as the p-contact layer. After the epitaxial layers had been grown, the sample was annealed in situ (700 °C, 15 min) under a N_2_ ambient to activate the Mg dopants. Figure 1b provides a transmission electron microscopy (TEM) image of the 293 nm UVB-LED structure; one of insets displays the MQW and p-layer structures, while the other displays the AlN layer and superlattice structures. The EDX mapping images of the insets of Figure 1b revealing the variable composition of Al and Ga are as shown in Figure 1c. Furthermore, Figure 1d presents the secondary ion mass spectroscopy (SIMS) depth profiles of the concentrations of the Mg and Si atoms, measured in units of atoms cm^–3^; the intensities of the Al and Ga atoms are provided in units of counts per second (cps). The concentration of Si atoms was approximately 1 × 10^19^ cm^–3^ in the Si-doped n-Al_0.5_Ga_0.5_N layer; in the CSL of the Si-doped Al_0.47_Ga_0.53_N and the MQWs, it decreased in steps to approximately 5.5 × 10^18^ and 5 × 10^18^ cm^–3^, respectively. The concentration of Mg atoms increased slightly at the interface between the undoped Al_0.45_Ga_0.55_N QB and the EBL, then remained constant at approximately 1 × 10^19^ cm^–3^ in the Mg-doped p-Al_0.55_Ga_0.45_N layer of the EBL. In contrast, in the layer of Mg-doped p-AlGaN with a graded Al content, the concentration of Mg atoms increased gradually to approximately 5 × 10^19^ cm^–3^, accompanied by a decrease in the Al content. Finally, the concentration of Mg atoms in the p-contact layer was approximately 7 × 10^19^ cm^–3^. These SIMS data suggest the effective incorporation of Mg atoms when using the designed p-type structure and growth conditions. LED chips were fabricated using standard flip-chip processing technologies. Mesa structures were defined through inductively coupled plasma etching to expose the n-Al_0.5_Ga_0.5_N layer surface. The n-contacts of Ti/Al/Ti/Au (100/200/30/100 nm) were deposited through electron beam evaporation and subjected to rapid thermal annealing (980 °C, 60 s). To form the transparent p-contact, a layer of indium tin oxide (ITO) that was 50 nm in thickness was sputtered on top of a p-GaN layer with a contact area of approximately 0.079 mm^2^ and then annealed (600 °C, 10 min). The LED chips were completed through the deposition of Ti/Pt/Au (50/30/100 nm) and a AuSn alloy that was 3 μm in thickness, functioning as bonding pads. The processed wafer was then lapped and scribed into the dimensions of 350 × 450 μm^2^.

The prepared 293 nm UVB-LED chips were flip-bonded using the eutectic method onto AlN-based direct plating copper ceramic (AlN-DPC) lead frames (LFs). The packaged samples were soldered onto an Al metal-core printed circuit board (MCPCB). The dimensions of the AlN-DPC LF and MCPCB were 3.75 (L) × 3.75 (W) × 1.4 (H) mm and 20 × 1.6 mm, respectively. The light output power (LOP), current–voltage characteristics, and EL spectra of the samples were measured using a calibrated ATA-5000 LED photoelectric measurement system (Everfine) equipped with an integrating sphere that was 30 cm in diameter. During measurement, the temperature of the heat sink mounting the samples was controlled at 298, 318, 338, or 358 K, while the driving direct current was varied at 10, 30, 50, 70, or 90 mA (current densities, approximately 12.7, 38.0, 63.6, 88.6, and 113.9 A cm^–2^, respectively.). To minimize the effect of self-heating of the chip, the stop interval was set to 5 min during each continuous wave (CW) measurement.

## 3. Results and Discussion

**Figure 2:**
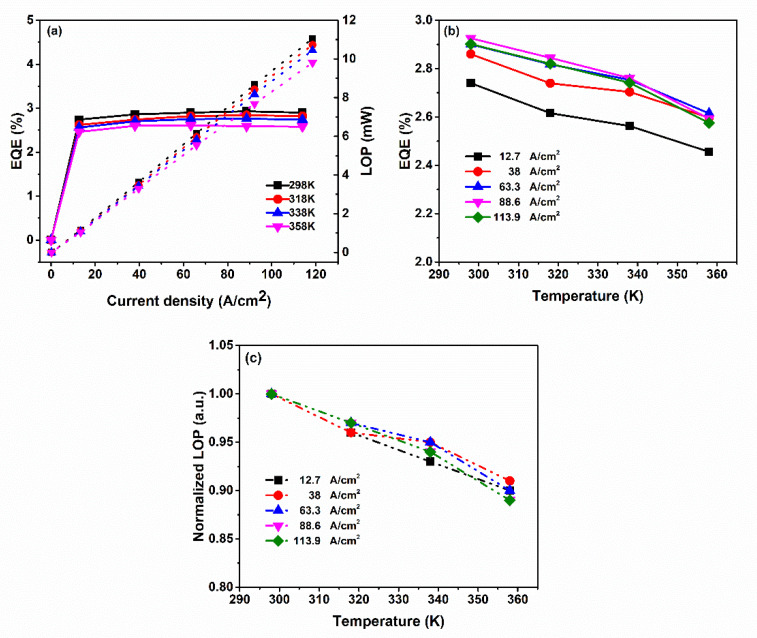
(**a**) EQEs (solid lines) and LOPs (dotted lines) of the 293 nm AlGaN-based UVB-LED plotted with respect to the forward current and measured at four different temperatures. (**b**) T-droop plotted with respect to temperature and measured at various forward currents. (**c**) Normalized LOPs plotted with respect to temperature and measured at various forward currents.

Figure 2a reveals the dependence of the EQEs and LOPs of the fabricated 293 nm UVB-LED on the four tested temperatures and various forward current densities. The LOP increased monotonically upon increasing the forward current density (*J*_f_) to 113.9 A cm^–2^, reaching maximum values of 11.0, 10.7, 10.4, and 9.8 mW at 298, 318, 338, and 358 K, respectively. The maximum EQEs at 298, 318, and 338 K (2.93, 2.84, and 2.76%, respectively) occurred when the value of *J*_f_ was 88.6 A cm^–2^. At 358 K, the maximum EQE was 2.61% when the value of *J*_f_ was 63.3 A cm^–2^; at 113.9 A cm^–2^, the maximum EQEs measured at 298, 318, 338, and 358 K decreased to 2.90, 2.82, 2.74, and 2.58%, respectively. In other words, we obtained very low J-droops of 0.83, 0.86, 0.70, and 1.52% at 298, 318, 338, and 358 K, respectively.

For a given value of *J*_f_, the EQEs decreased monotonically upon increasing the temperature, but the tendency upon increasing the value of *J*_f_ was not obvious. Figure 2b presents the T-droops plotted with respect to temperature. The T-droops measured at values of *J*_f_ of 12.7, 38.0, 63.3, 88.6, and 113.9 A cm^–2^ were 10.38, 9.19, 9.83, 11.37, and 11.25%, respectively. We observed monotonically increasing T-droops upon increasing the temperature from 298 to 358 K, but the maximum T-droop of 11.37% occurred when the value of *J*_f_ was 88.6 A cm^–2^. Moreover, Figure 2c displays the normalized LOPs with respect to temperature measured at various values of *J*_f_. The decays in the normalized LOPs were approximately 10% upon increasing the temperature from 298 to 358 K at values of *J*_f_ of 12.7, 38.0, 63.3, 88.6, and 113.9 A cm^–2^. These findings suggest that mechanism through which the J-droop was overcome was related to an enhancement in the uniform distribution of the concentrations of injected electrons and holes within the MQWs. Through our subtle design of the p-type AlGaN-based layer, with its optimized composition and doping level, the hole injection efficiency increased, and the efficiency droop was inhibited for values of *J*_f_ of up to 113.9 A cm^–2^.

In contrast, Figure 2b reveals that a higher T-droop did not occur at higher values of *J*_f_. In other words, the T-droop did not increase monotonically upon increasing the value of *J*_f_. This behavior is inconsistent with previous findings of the T-droop increasing or decreasing upon increasing the current at a constant temperature [36,43]. Moreover, the normalized LOP decreased upon increasing the temperature from 298 to 358 K (Figure 2c). The decay rate of the LOP was slightly faster when increasing the value of *J*_f_ for temperatures in the range from 338 to 358 K. After applying the equation of the characteristic temperature [35],

*L*(*T*) = *L*(0 K) exp^(−*T*/*T*c)^ (1)

we estimated the minimum characteristic temperatures (*T*_c_) to be approximately 454 K at a value of *J*_f_ of 12.7 A cm^–2^ and approximately 749 K at a value of *J*_f_ of 63.3 A cm^–2^. We suggest that the mechanism of T-droop in this study arose from the uniform distribution of the concentrations of injected electrons and holes within the MQWs, even at the relatively high temperature of 358 K. Through the subtle design of the p-type AlGaN-based layer, with its optimized composition and doping level, high values of *T*_c_ were achieved while inhibiting carrier leakage.

**Figure 3:**
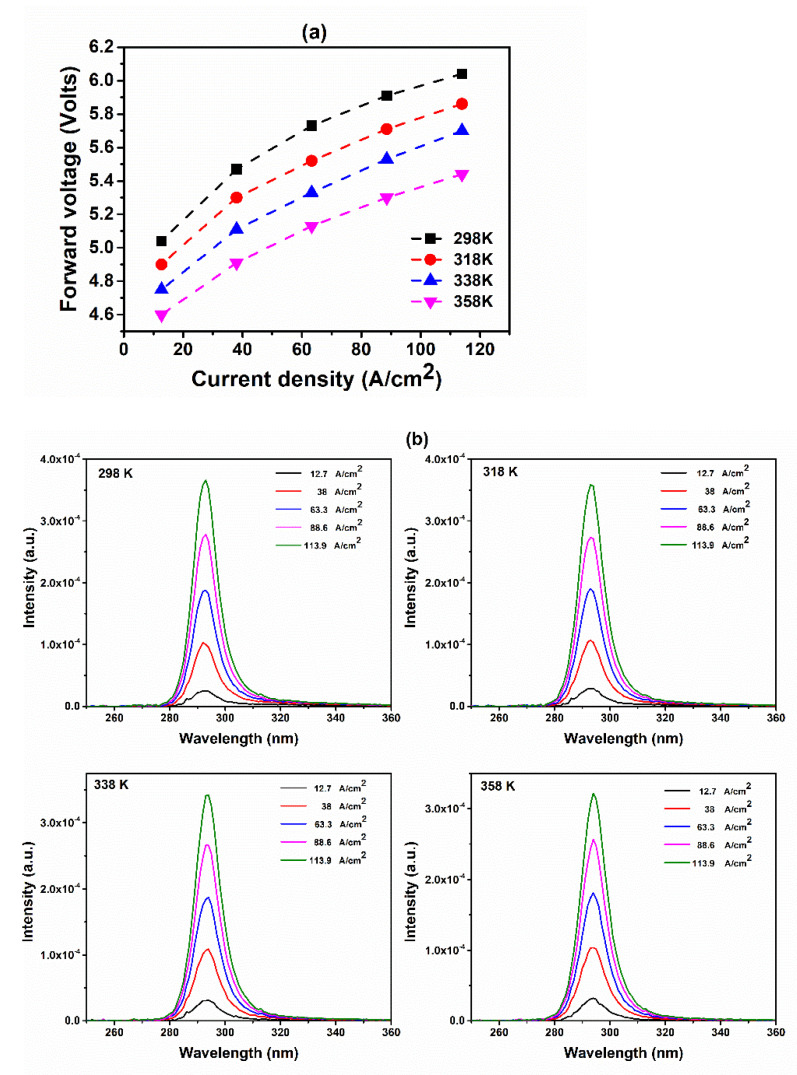
(**a**) Forward voltages and (**b**) EL spectral data of the 293 nm UVB-LED recorded at various forward currents and temperatures of 298, 318, 338, and 358 K.

Figure 3a,b display the forward voltages (*V*_f_) and EL spectral data recorded at various forward currents and temperatures of 298, 318, 338, and 358 K. The forward voltage decreased, and the peak wavelength increased upon increasing the temperature at the same forward current due to the shrinking of the band gap; the peak wavelength underwent a slight blue shift (<0.5 nm) upon increasing the current density from 12.7 to 63.3 A cm^–2^ when the temperature was 298 or 318 K. This result implies the absence of a band-filling effect or coulomb screening of the quantum confinement Stark effect (QCSE) in the MQWs of the fabricated 293 nm UVB-LED. When the temperature was 338 or 358 K, the peak wavelength underwent a slight red shift upon increasing the current from 63.3 to 113.9 A cm^–2^ due to the effect of self-heating. Thus, the temperature-dependent recombination mechanism in the MQWs of the fabricated 293 nm UVB-LED was suppressed at temperatures ranging from 298 to 358 K.

**Figure 4:**
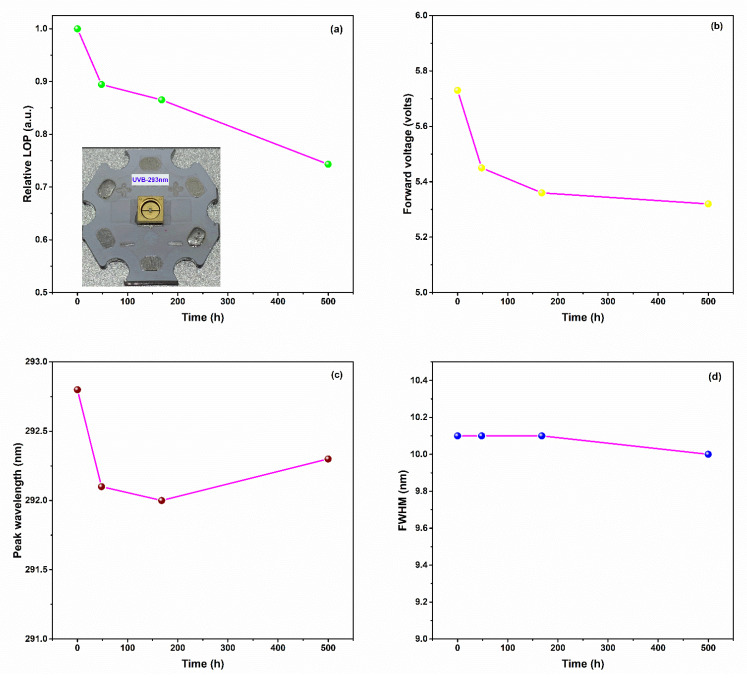
(**a**) Relative LOPs of the 293 nm UVB-LED. Inset: photograph of the UVB-LED on MCPCB. (**b**) Forward voltages, (**c**) EL peak wavelengths, and (**d**) EL FWHMs of the AlGaN-based 293 nm UVB-LED measured at a value of *J*_f_ of 63.3 A cm^–2^ during the aging tests.

Figure 4a presents the relative LOPs measured over time for the 293 nm UVB-LED operated at a forward current of 50 mA (current density: ca. 63.3 A cm^–2^) at room temperature as well as a photograph of the UVB-LED on MCPCB. Rapid degradation of the LOP, to 89.4% of its initial value, occurred in the first 48 h of the aging operation; it then decreased to 86.5% of its initial value when the aging time reaching 168 h. After an aging time of 500 h, the LOP decreased to 74.3% of its initial value. Such behavior has been observed many times previously [38,39,40,41,42,43]. The LOP decreased more slowly after 500 h of aging operations, in accordance with previous reports [40,43]. This performance is close to the specifications of commercial solid-state lighting products, with the estimated 70% lifetime (L70) being comparable with those reported for a 310 nm UVB LED operated at current densities of 28.5 and 33.5 A cm^–2^ [38,43]. Figure 4b–d present the time-dependence of the forward voltage, the EL peak wavelength, and the EL FWHM, respectively, all measured during the aging tests performed at a value of *J*_f_ of 63.3 A cm^–2^. The forward voltage decreased dramatically at the onset of operation but remained stable during operation between 168 and 500 h. This behavior is very similar to that reported previously [40,43]. Interestingly, while the LOPs and forward voltages decreased simultaneously upon increasing the aging time to 500 h, no significant changes occurred in the peak wavelengths or in the FWHMs. This behavior suggests that the crystal quality of the MQWs did not undergo serious deterioration over time.

## 4. Conclusions

We prepared an AlGaN-based UVB-LED that operated nearly free of efficiency droop at its peak wavelength of 293 nm at temperatures ranging from 298 to 358 K. The maximum EQEs measured at a current density of 88.6 A cm^–2^ at 298, 318, and 338 K were 2.93, 2.84, and 2.76%, respectively. Nevertheless, the J-droops measured at 298, 318, and 338 K were all less than 1%. When the temperature was 358 K, the maximum EQE (2.61%) occurred at a current density of 63.3 A cm^–2^, and the J-droop was 1.52%. We suspect that the main mechanism responsible for overcoming the J-droop was the uniform distribution of the concentrations of the injected electrons and holes within the MQWs. Through the subtle design of the p-type AlGaN layer, which had an optimized composition and doping level, we increased the efficiency of hole injection and inhibited the Auger recombination mechanism, as determined experimentally. After aging for 500 h at a value of *J*_f_ of 63.3 A cm^–2^, no significant changes occurred in the peak wavelengths or the FWHMs. This behavior suggests that the crystal quality of the MQWs was not impacted significantly over time.

## Data Availability

Not applicable.

## Sample Availability

Samples of the compounds ... are available from the authors.
